# Post-treatment Method for the Synthesis of Monodisperse Binary FePt-Fe_3_O_4_ Nanoparticles

**DOI:** 10.1186/s11671-017-2312-5

**Published:** 2017-09-19

**Authors:** Zhilu Liu, Chun Wu, Liang Niu, Ganting Yang, Kai Wang, Wenli Pei, Qiang Wang

**Affiliations:** 1 Key Laboratory of Electromagnetic Processing of Materials (Ministry of Education), Northeastern University, Shenyang, 110819 People’s Republic of China; 2 School of Materials Science and Engineering, Northeastern University, Shenyang, 110819 People’s Republic of China; 3 Key Laboratory of Anisotropy and Texture of Materials (Ministry of Education), Northeastern University, Shenyang, 110819 People’s Republic of China

**Keywords:** FePt nanoparticles, Fe_3_O_4_ nanoparticles, Excess iron, Grain size, Oxidize, Post-treatment method

## Abstract

To obtain the optimal 1:1 composition of FePt alloy nanomaterials by polyol synthesis, the iron precursor (iron pentacarbonyl, Fe(CO)_5_) must be used in excess, because the Fe(CO)_5_ exists in the vapor phase at the typical temperatures used for FePt synthesis and cannot be consumed completely. Fabrication of Fe_3_O_4_ nanoparticles by consuming the excess iron precursor was an effective strategy to make full use of the iron precursor. In this paper, a facile post-treatment method was applied to consume the excess iron, which was oxidized to Fe_3_O_4_ after post-treatment at 150 and 200 °C, and a monodisperse binary FePt-Fe_3_O_4_ nanoparticle system was generated. The post-treatment method did not affect the crystal structure, grain size, or composition of the FePt nanoparticles. However, the content and grain size of the fcc-Fe_3_O_4_ nanoparticles can be increased simply by increasing the post-treatment temperature from 150 to 200 °C.

## Background

FePt nanomaterials attract considerable attention owing to their promising applications in the fields of magnetic storage, permanent magnets, fuel cell catalysis, and biomedicine [1–5]. A polyol method, which involves thermal decomposition of iron pentacarbonyl (Fe(CO)_5_), reduction of platinum acetylacetonate (Pt(acac)_2_), and stabilizing through surfactants oleic acid (OA) and oleylamine (OAm), has been widely used to synthesize FePt nanomaterials. This method has many advantages, including its facile synthesis, economical approach, and potential for mass production [6]. In general, the performance of FePt nanomaterials strongly depends on their composition [7–9] To obtain the optimal 1:1 ratio of Fe:Pt, the Fe precursor must be used in excess (at twice the amount of the Pt precursor) because Fe(CO)_5_ exists in the vapor phase at the typical temperatures used for FePt synthesis and cannot be consumed completely [6]. Many researchers have studied the form that the excess iron takes and have tried to make full use of the iron precursor. It was reported that the rest of Fe(CO)_5_ could react with OA or OAm to form the Fe-oleate or Fe(CO)_x_-OAm complex [10, 11]. Increasing the synthesis temperature is a promising strategy for consuming the excess irons and generating Fe_3_O_4_ in the reflux process. [12] The entire iron precursor could be consumed when the synthesis temperature increased to 300 °C, the iron atoms nucleated and grew on the FePt nanoparticles to produce dumbbell-like nanostructures when the molar ratio of the Fe and Pt precursors was equal to 3 [12]. At 280 °C and a molar ratio of 2.2, the excess iron formed a very thin Fe_3_O_4_ shell on the FePt nanoparticles [13]. Otherwise, oxidation under air also could be applied to ensure the formation of Fe_3_O_4_ [14]. In brief, fabrication of Fe_3_O_4_ nanoparticles by consuming the excess iron precursor was an effective strategy to make full use of the iron precursor, because the self-assembly of FePt and Fe_3_O_4_ nanoparticles was a permission method to fabricate high-performance exchange-coupled nanocomposites magnets [2].

Here, we report another facile post-treatment method to consume the excess iron. A monodisperse binary FePt-Fe_3_O_4_ nanoparticle system was generated, and the influence of the post-treatment temperature on the content and size of the Fe_3_O_4_ nanoparticles was studied.

## Methods

Excess iron was consumed, and monodisperse binary FePt-Fe_3_O_4_ nanoparticles were synthesized by post-treatment of a FePt-hexane system. The apparatus and method used for the synthesis of the FePt nanoparticles was described in our previous research [15]. In brief, 0.1 mmol Pt(acac)_2_ and 1.0 mmol Fe(CO)_5_ were used as precursors, 1.6 mL OA and 2 mL OAm were applied as surfactants, and 10 mL dibenzyl ether (DE) was acted as the solvent. The FePt nanoparticles were synthesized by maintaining this mixture at 175 °C for 1 h under a high-purity Ar atmosphere to prevent oxidation. The particles were washed repeatedly with ethanol, were centrifuged, and were finally dispersed in hexane at a concentration of about 5 mg/mL. In a typical post-treatment process, 2 mL of the as-synthesized FePt-hexane solution and 2 mL OAm were injected into a quartz crucible, which was placed inside a vertical tubular resistance furnace [16]. Then, the quartz crucible was heated to 150 or 200 °C at a rate of 5 °C/min, and held at that temperature for 1 h without a protective atmosphere. After cooling, the post-treated nanoparticles were washed, centrifuged, and stored in hexane.

Samples for transmission electron microscopy (TEM, JEM-2100F) analysis were prepared by drying a dispersion of the nanoparticles on amorphous carbon-coated copper grids. The nanoparticles size and their distribution were collected through counting at least 100 particles in TEM images by using Win Roof software. The crystal structure was determined by selected area electron diffraction (SAED) and X-ray diffraction (XRD) using an Ultima IV instrument. To quantitatively analyze the weight percentage of FePt-phase and Fe_3_O_4_-phase in the monodisperse binary FePt-Fe_3_O_4_ nanoparticle system, a standard Rietveld method was applied to fit the XRD patterns. The composition of the nanoparticles was analyzed by TEM-associated energy dispersive spectroscopy (EDS) and X-ray photoelectron spectroscopy (XPS, ESCALAB250). The XPS samples were prepared by drying nanoparticle-hexane ink on a Si substrate in air. The magnetic properties were measured by vibrating sample magnetometer (VSM) at room temperature on a MicroSense EZ9 magnetometer.

## Results and Discussion

**Fig. 1:**
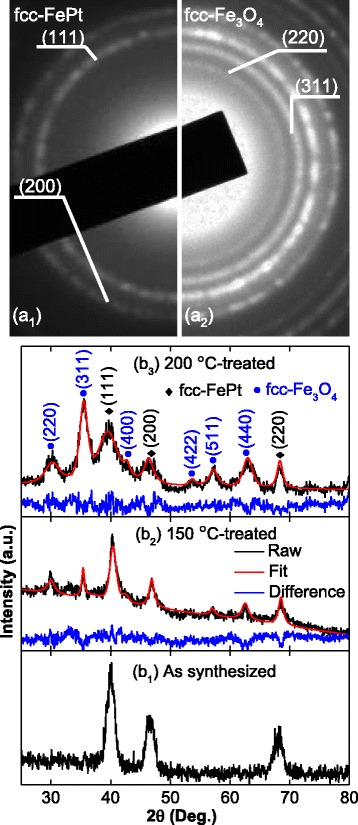
Selected area electron diffraction patterns of as-synthesized (a_1_) and 200 °C post-treated (a_2_) nanoparticles. X-ray diffraction patterns of as-synthesized and post-treated nanoparticles ((b_1_) as-synthesized; (b_2_) 150 °C post-treated; (b_3_) 200 °C post-treated)

A typical SAED pattern of the as-synthesized FePt nanoparticles is shown in Fig. 1(a_1_); it was indexed as rings of fcc-FePt (111) and (220) faces. The SAED pattern after post-treatment at 200 °C is shown in Fig. 1(a_2_). There are clearly two different rings in the post-treated samples; one originates from fcc-Fe_3_O_4_ (200) and the other from (311). The XRD patterns of the as-synthesized and post-treated nanoparticles are shown in Fig. 1(b_1_–b_3_). The diffraction peaks of the as-synthesized FePt nanoparticles are indexed as a disordered fcc-phase (Fig. 1b_1_), which agrees well with the SAED results and those of other studies [6, 15]. The diffraction peak intensities of the fcc-Fe_3_O_4_ phase increased when the temperature was increased from 150 °C to 200 °C. As reported previously [12], the intensity of the peaks in the XRD patterns depends on the content of fcc-Fe_3_O_4_. To quantitatively analyze the weight percentage of the fcc-Fe_3_O_4_ in the post-treated nanoparticles, a standard Rietveld method was applied to fit the patterns. In Fig. 1 (b_2_) and (b_3_), the red lines are the fitted patterns and the blue lines are the difference patterns between the raw and fitted patterns. Clearly, the fitted patterns agreed well with the measured patterns (black line), the fcc-Fe_3_O_4_ content increases from 42.6 to 82.9 wt.% when the post-treated temperature rises from 150 to 200 °C. These results indicate that the excess iron is oxidized to fcc-Fe_3_O_4_ nanoparticles during post-treatment, and the content of the fcc-Fe_3_O_4_ phase increased when the post-treatment temperature was further increased to 200 °C.

**Fig. 2:**
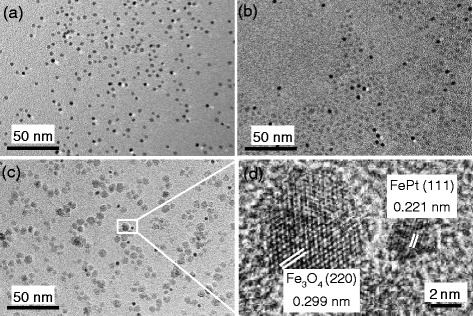
Transmission electron microscopy (TEM) images of as-synthesized nanoparticles (**a**), and nanoparticles after post-treatment at 150 °C (**b**) and 200 °C (**c**). **d** High-resolution TEM image of the area inside the white box in (**c**)

Fig. 2 shows the TEM images of the as-synthesized and post-treated nanoparticles. In Fig. 2a, the as-synthesized FePt nanoparticles are black and monodisperse. After post-treatment at 150 °C, as shown in Fig. 2b, the nanoparticles remain monodisperse and do not aggregate; it is notable that some gray particles are observed. When the post-treatment temperature was increased to 200 °C (Fig. 2c), the observed nanoparticles are combination of black and gray particles still. However, the size of the gray nanoparticles is larger than that of the gray nanoparticles post-treated at 150 °C. A high-resolution TEM (HRTEM) image of the nanoparticles in the white box of Fig. 2c is shown in Fig. 2d. The distance between the lattice fringes in the gray nanoparticles is 0.299 nm, which corresponds to the lattice spacing of fcc-Fe_3_O_4_ (200). The interfering distance in the black nanoparticles is approximately 0.221 nm, which corresponds to the lattice spacing of fcc-FePt (111). The TEM and XRD results thus indicate that the black nanoparticles are fcc-FePt and the gray nanoparticles are fcc-Fe_3_O_4_. The light-and-shade contrast of FePt and Fe_3_O_4_ nanoparticles is different in the TEM images, and is similar to that of the dumbbell-like FePt-Fe_3_O_4_ nanostructure [12]. The monodisperse gray Fe_3_O_4_ nanoparticles could be found only in the post-treated samples, this means the post-treatment method would not induce the aggregate of nanoparticles and it is an effective way of producing the monodisperse binary FePt-Fe_3_O_4_ nanoparticle system.

**Fig. 3:**
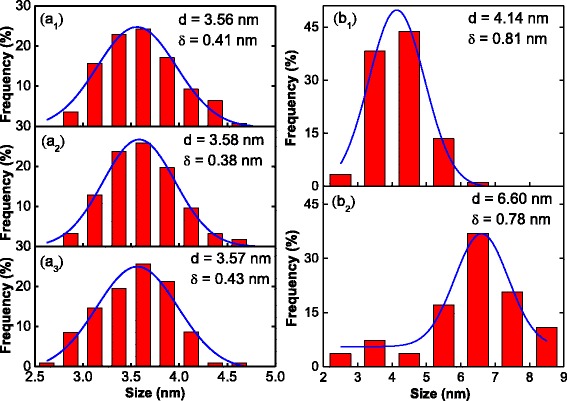
Grain size distribution of black FePt nanoparticles ((a_1_) as-synthesized; (a_2_) 150 °C post-treated; (a_3_) 200 °C post-treated) and gray Fe_3_O_4_ nanoparticles ((b_1_) 150 °C post-treated; (b_2_) 200 °C post-treated)

To quantitative analysis, the effects of post-treatment temperature on the growth of FePt and Fe_3_O_4_ nanoparticles, the grain size of nanoparticles produced in different situation was counted. The grain size distribution of black FePt nanoparticles is shown in Fig. 3(a_1–_a_3_), there are accord well with the Gauss function and locate in the same range. The average grain size of FePt nanoparticles is 3.56 ± 0.41, 3.58 ± 0.38, and 3.57 ± 0.43 nm for the as-synthesized, 150 °C post-treated and 200 °C post-treated samples, respectively. The grain size of all the black FePt nanoparticles is close to 3.6 nm, which indicates that the post-treatment method does not markedly influence the grain size of the FePt nanoparticles. However, the grain size of gray Fe_3_O_4_ nanoparticles increased from 4.14 ± 0.81 nm (Fig. 3(b_1_)) to 6.60 ± 0.78 nm (Fig. 3(b_2_)) when the post-treatment temperature increased from 150 to 200 °C. As the monodisperse FePt and Fe_3_O_4_ spherical nanoparticles are uniformly distributed (as shown in Fig. 2), the volume fraction of Fe_3_O_4_ in the binary FePt-Fe_3_O_4_ nanoparticle system has been counted through at least five different zones. The result shows that the volume fraction of Fe_3_O_4_ increases from 64.3 ± 9.7% to 92.5 ± 6.1% when the post-treated temperature rises from 150 to 200 °C, which is essentially in agreement with the weight percentage of XRD results. This means that tuning the post-treatment temperature is an effective way of controlling the growth of the excess iron and the grain size of the Fe_3_O_4_ nanoparticles in the monodisperse binary FePt-Fe_3_O_4_ nanoparticle system.

**Fig. 4:**
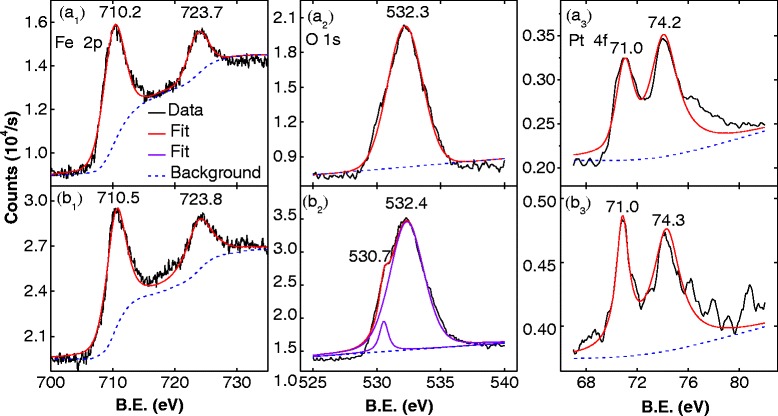
X-ray photoelectron spectra from as-synthesized nanoparticles ((a_1_): Fe 2p, (a_2_): O 1s, (a_3_): Pt 4f) and nanoparticles post-treated at 200 °C ((b_1_): Fe 2p, (b_2_): O 1s, (b_3_): Pt 4f)

Figure 4 shows the XPS analysis of the as-synthesized nanoparticles and those treated at 200 °C. The Fe 2p signal is composed of Fe 2p_3/2_ and Fe 2p_1/2_, and the binding energies of these two peaks in the as-synthesized FePt nanoparticles were 710.2 and 723.7 eV, respectively (Fig. 4(a_1_)). These values are higher than those of pure Fe (710 and 723 eV) because of the bonding between Fe and Pt in a single cell [13]. After post-treatment at 200 °C, the Fe 2p binding energy increased to 710.5 and 723.8 eV, as shown in Fig. 4(b_1_); this is closer to the values for Fe_3_O_4_ (710.6 and 724.1 eV) [16]. The O 1 s binding energy of the as-synthesized FePt nanoparticles was 532.3 eV (Fig. 4(a_2_)), which corresponds to absorbed H_2_O or O_2_ at the surface. Another O 1 s peak at 530.7 eV was found in the samples post-treated at 200 °C (Fig. 4(b_2_)), which was ascribed to the O^2−^ ions resulting from the oxidation of Fe [13]. No satellite peaks were observed in the Fe 2p spectra, which indicate that the Fe is in the Fe_3_O_4_, not the Fe_2_O_3_ [17]. This is consistent with the XRD and TEM results. The XPS spectrum of Pt for the as-synthesized and 200 °C-treated sample is shown in Fig. 4(a_3_) and Fig. 4(b_3_). The Pt 4f region of XPS spectra was characterized by a typical spin-orbit doublet (4f_7/2_ and 4f_5/2_); their binding energy are close to 71.0 and 74.3 eV, respectively. The post-treatment method has no effect on the binding energy of Pt 4f.

**Fig. 5:**
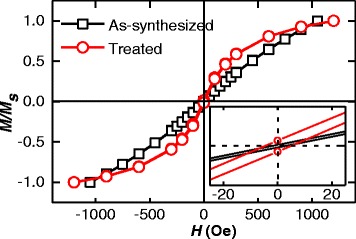
Room-temperature magnetic hysteresis loop loops of as-synthesized and 200 °C-treated nanoparticles

The room-temperature (298 K) magnetic hysteresis loop loops of as-synthesized FePt nanoparticles and 200 °C-treated binary FePt-Fe_3_O_4_ nanoparticle is shown in Fig. 5. The magnetic hysteresis loop of as-synthesized FePt nanoparticles is linear and its coercive is closed to zero, which indicates the FePt nanoparticles are superparamagnetic at room-temperature. As reported earlier, the disordered fcc structure and the smaller grain size would lead to the superparamagnetic behavior of FePt nanoparticle [13]. The very small but nonzero coercivity (5.7 Oe) is observed for the monodisperse binary FePt-Fe_3_O_4_ nanoparticle at room-temperature. Normally, the Fe_3_O_4_ nanoparticle is superparamagnetic when the grain size is smaller than 20 nm, [18] some researchers also found that the coercivity of Fe_3_O_4_ nanoparticles constant at about 5 Oe in the range of 8 to 15 nm [19]. In this research, the monodisperse binary FePt-Fe_3_O_4_ system is combined of 17.1 wt.% 3.6 nm FePt nanoparticle and 82.9 wt.% 6.6 nm Fe_3_O_4_ nanoparticle, the interaction between those two kinds of different nanoparticles maybe also lead to the nonzero coercivity result. The zero coercivity FePt nanoparticle transforms to nonzero after post-treatment at 200 °C, which proves once again that the Fe_3_O_4_ nanoparticle are generated by using the post-treatment method.

The Fe/Pt ratio in the as-synthesized and 200 °C-treated samples could be calculated trough the peaks of Fe 2p and Pt 4 f in Fig. 4. The analysis revealed that the Fe content in XPS samples (nanoparticle-hexane ink) were 88.6 and 90.5%, respectively. However, the TEM-EDS results indicate that the Fe counts in the FePt nanoparticles from as-synthesized and post-treated were nearly the same (72.8 and 72.3%), and lower than the Fe counts in the FePt-hexane ink and the binary FePt-Fe_3_O_4_ nanoparticle system. We therefore deduced that the excess iron transformed from vapor to liquid (into the FePt-hexane ink) in the reflux, cooling, and washing processes during the synthesis of the FePt nanoparticles. The nature of the excess iron species in the FePt-hexane ink is still unclear, but it is most likely that they are combined with surfactants to ensure the stability of the FePt nanoparticles [10, 11]. The oxidation of the excess irons, or the growth Fe_3_O_4_ nanoparticles, is strongly dependent on the temperature and atmosphere. Under the high-purity argon system, the Fe_3_O_4_ nanoparticle cannot be obtained at various temperatures. And the FePt-solution would dry out even at 100 °C under the vacuum environment. It is facile to obtained monodisperse binary FePt-Fe_3_O_4_ nanoparticle system in air, the Fe_3_O_4_ nanoparticle is generated when the temperature is above 100 °C, however, if the temperature is as high as 250 °C, the FePt-solution would be dry out also. The grain size and content of Fe_3_O_4_ nanoparticle in the binary FePt-Fe_3_O_4_ nanoparticle system are increased when the post-treatment temperature increases from 150 to 200 °C, which would be caused by the temperature enhanced diffusion growth of irons in FePt-hexane-OAm solution.

## Conclusions

In summary, the post-treatment method is an effective strategy for the consumption of excess iron used in the polyol synthesis of FePt nanomaterials. The excess iron is oxidized to Fe_3_O_4_ after post-treatment, and a monodisperse binary FePt-Fe_3_O_4_ nanoparticle system is generated. The content and grain size of the fcc-Fe_3_O_4_ nanoparticles can be increased facilely by increasing the post-treatment temperature from 150 to 200 °C.
